# Novel Approach for Treating Diabetes in a Patient With the Heterozygous Pathogenic Variant R46Q in the Insulin Gene

**DOI:** 10.1210/jcemcr/luae134

**Published:** 2024-07-18

**Authors:** Kristina Laugesen, Søren Gregersen, Julie Støy

**Affiliations:** Department of Clinical Biochemistry, Aarhus University Hospital, 8200 Aarhus, Denmark; Steno Diabetes Center Aarhus, Aarhus University Hospital, 8200 Aarhus, Denmark; Department of Clinical Medicine, Aarhus University, 8000 Aarhus, Denmark; Steno Diabetes Center Aarhus, Aarhus University Hospital, 8200 Aarhus, Denmark; Department of Clinical Medicine, Aarhus University, 8000 Aarhus, Denmark

**Keywords:** monogenetic diabetes, maturity-onset diabetes of the young, MODY, insulin gene mutation, metformin, sodium-glucose cotransporter 2 inhibitor

## Abstract

Maturity-onset diabetes of the young (MODY) is a monogenic disorder of glucose homeostasis with several subtypes, each defined by a distinct genetic etiology. Heterozygous pathogenic variants in the insulin gene are rare causes of MODY, and optimal treatment strategies remain uncertain. Herein we describe a patient with diabetes caused by the heterozygous pathogenic variant R46Q in the insulin gene and the glycemic response to selected antidiabetic treatment regimens. The R46Q pathogenic variant leads to secretion of both mutant and wild-type insulin. In vitro, the mutant insulin is associated with a lower insulin-receptor affinity compared with wild-type insulin and a decline in wild-type insulin secretion. In our patient, treatment with a combination of long- and short-acting insulin led to a decline in hemoglobin A1C (HbA1c), although not to the recommended target. A shift to metformin and subsequent add-on of a sodium-glucose cotransporter 2 inhibitor (SGLT2i) resulted in HbA1c levels of less than 7% (53 mmol/mol) and durable glycemic control. Continuous glucose monitoring and oral glucose tolerance tests confirmed that treatment with metformin and SGLT2i was superior to treatment with insulin. In conclusion, diabetes caused by the pathogenic variant R46Q in the insulin gene may be effectively treated with noninsulin.

## Introduction

Maturity-onset diabetes of the young (MODY) is a monogenic disorder of glucose homeostasis accounting for an estimated 1% to 2% of all people with diabetes (1). Several subtypes have been identified, each defined by a distinct genetic etiology. In contrast to other forms of diabetes, MODY often exhibits autosomal dominant inheritance, but de novo mutations occur as well (1). MODY often presents in childhood or early adulthood, and in the majority of patients, the diabetes is well controlled with diet and/or oral antidiabetic agents. For the common subtypes of MODY (HNF1A-MODY, HNF4-MODY, and GCK-MODY), the associated phenotypes and responses to different treatment regimens have been described in observational studies, case series, and a few clinical trials.

Heterozygous missense mutations in the insulin gene are a common cause of permanent neonatal diabetes, another monogenic form of diabetes, but are occasionally identified in patients with MODY phenotypes. No randomized clinical trials or published case series inform how these patients are best treated. In 2017, we reported a detailed characterization of the pathophysiology of a specific insulin gene pathogenic variant (R46Q) identified in a patient with MODY (2). In the present case report, we describe how this patient responded to different treatment regimens and propose that patients carrying this pathogenic variant or others resulting in similar pathophysiology might respond favorably to noninsulin treatment regimens.

## Case Presentation

In 2011, a healthy female of Danish–Indian ancestry was screen-detected with diabetes at age 15. At time of diagnosis, her hemoglobin A1C (HbA1c) was 6.5% (48 mmol/mol, diagnostic cut off is ≥48 mmol/mol), fasting C-peptide was 320 pmol/L (normal range: 210-1150 pmol/L), fasting p-glucose was 128 mg/dL (7.1 mmol/L, diagnostic cut off is ≥7.0 mmol/L), and antibodies against glutamic acid decarboxylase 65 were negative (2). Her body mass index was 17 kg/m^2^, and she had normal blood pressure and lipids. She had a family history of diabetes (impaired fasting glucose from age 50 in her Indian father and paternal uncle and type 2 diabetes in her paternal grandparents) (2).

## Diagnostic Assessment

Because of clinical suspicion of MODY, the initial diagnostic workup included genetic testing for MODY. She was found to be a heterozygous carrier of a nonsynonymous pathogenic variant in the insulin gene R46Q (c.137G > A). The pathogenic variant was de novo in origin (the parents were not carriers) and had previously been identified in other families affected by diabetes (3-5). This specific pathogenic variant leads to an amino acid substitution at residue 22 of the B-chain of preproinsulin and is also referred to as GlnB22-insulin (2, 6). In our previous examination of the patient, we found that both GlnB22-insulin and wild-type insulin were secreted in a glucose-dependent manner, but concentrations of GlnB22-insulin were found to be 1.5 times higher than wild-type insulin at all times during a standard 75 g oral glucose tolerance test (OGTT) (2). In vitro studies showed that the insulin-receptor affinity of GlnB22-insulin was only 57% of that of wild-type insulin (2, 6). Further, heterozygous expression of R46Q-insulin in INS-1 cells was associated with a decline in wild-type insulin secretion (2). Interestingly, in the in vitro model this mutation was not associated with increased expression of markers of endoplasmic stress, which is considered a hallmark of the pathophysiology of the insulin gene mutations found in patients with the severe phenotype of neonatal diabetes (2).

## Treatment

**Figure 1.**
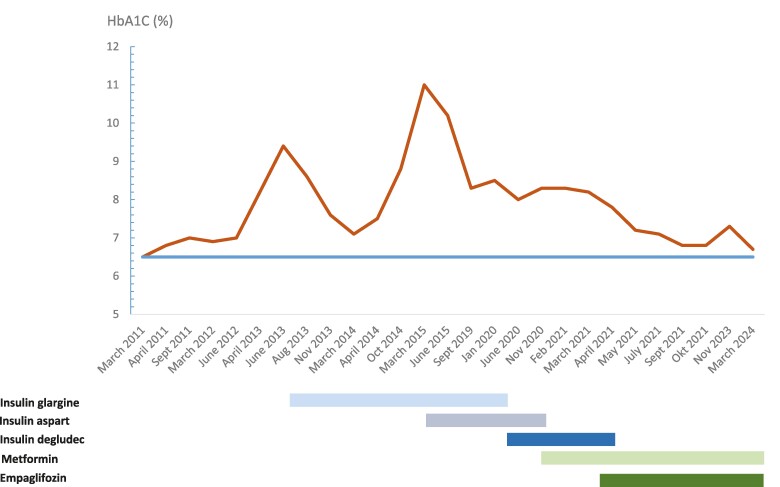
Timeline of treatment regimens and glycemic response based on hemoglobin A1c (%).Dosing was 14 to 28 U daily for insulin glargine, 8 to 22 U daily for insulin aspart, 8 to 24 U daily for insulin degludec, 1 g twice daily for metformin, and 10 mg once daily for empaglifozin.

Figure 1 shows a timeline for treatment regimens and responses in HbA1c. Following diagnosis in 2011, the patient sustained acceptable glycemic control (HbA1c ≤ 7% [53 mmol/mol]) with lifestyle interventions alone. In 2013, glycemic control deteriorated reaching a maximum HbA1c of 9.4% (79 mmol/mol). Thus, the patient started long-acting insulin glargine 14-28 U once daily. Reflecting further disease progression, HbA1C reached 11% (97 mmol/mol) in 2015 and short-acting insulin aspart 8-24 U was added. The combination of long- and short-acting insulin led to a decline in HbA1c, although not to the recommended target (Fig. 1). Compliance with glucose monitoring and insulin dosing was uncertain.

## Outcome and Follow-up

**Figure 2.**
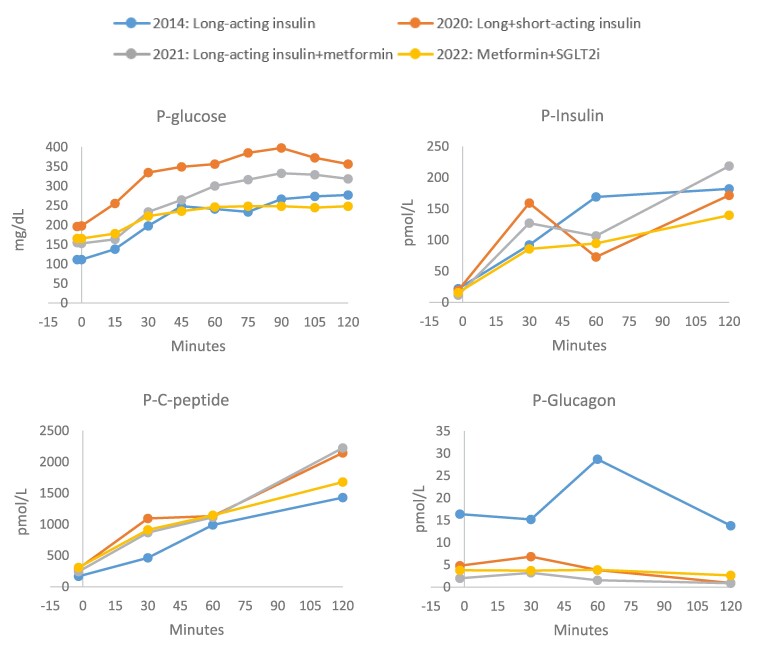
Oral glucose tolerance test during different treatment regimens. The patient fasted for 10 hours before each test. Insulin was paused for 72 hours before and oral antidiabetic treatment was paused on the same day.

In 2020, Riar and colleagues described a Canadian sibling pair with diabetes caused by the identical pathogenic variant (3). In this report, agents with beta cell stimulating effects, including a dipeptidylpeptidase-4 inhibitor, a sulfonylurea, and a glucagon-like peptide-1 receptor agonist were associated with progressive hyperglycemia, whereas treatment with metformin, decreasing the need for insulin, led to HbA1c of less than 6.5% (48 mmol/mol) after 6 months of treatment (3). In 2020, our patient had been diagnosed with diabetes for 9 years, and she had a normal body mass index of 22 kg/m^2^ and normal blood pressure and lipids. Following the report by Riar and colleagues (3), our patient was transferred from insulin therapy to metformin 1 g twice daily with subsequent add-on of a sodium-glucose cotransporter 2 (SGLT2) inhibitor (empagliflozin 10 mg once daily), resulting in HbA1c levels of less than 7% (53 mmol/mol) and durable glycemic response (Fig. 1). The treatment with metformin and empagliflozin was tolerated well by the patient. Continuous glucose monitoring was performed with Freestyle Libre sensor (ie, nonblinded and under free-living conditions) before initiation of metformin (December 2020), after initiation of metformin (February 2021), and after add-on of empagliflozin (June 2022). Time in range for glucose (70.2-180 mg/dL [3.9-10.0 mmol/L]) was 54% in 2020, 88% in 2021, and 95% in 2022 and glucose variation (expressed by percent coefficient variation) was 25.5%, 30.0%, and 17.6%, respectively. Further, a standard 75 g oral OGTT was performed during the different treatment regimens as shown in Fig. 2. Notably, glucose levels were lower at all times throughout the OGTT when treated with metformin (the 2 OGTTs in 2021/2022) vs treatment with long- and short-acting insulin (2020) (Fig. 2). Further, beta cell function remained relatively stable between 2011 (fasting C-peptide of 320 pmol/L and p-glucose of 128 mg/dL [7.1 mmol/L]) and 2022 (fasting of C-peptide 308 pmol/L and p-glucose of 166 mg/dL [9.2 mmol/L]) (Fig. 2).

## Discussion

The current case report provides insights into the management of individuals with diabetes caused by the heterozygous pathogenic variant R46Q in the insulin gene. As reported previously, metformin may play a key role in obtaining glycemic control in patients with this pathogenic variant (3). We add to the existing knowledge that SGLT2 inhibitors can contribute to further improvements of glycemic control when metformin in monotherapy is insufficient.

Heterozygous pathogenic variants in the insulin gene can cause monogenic diabetes through effects on the processing of proinsulin to insulin, beta cell function, and/or altered insulin-receptor affinity (2, 6, 7). The pathogenic variants lead to a broad spectrum of clinical phenotypes, ranging from severe neonatal diabetes with complete insulin deficiency to adult-onset diabetes with substantial beta cell function at diagnosis (7). Development of diabetes in patients with the heterozygous pathogenic variant R46Q is likely explained by a combination of lower receptor binding affinity of the secreted GlnB22-insulin and some degree of impaired beta cell function (2, 6). Still, in contrast to neonatal diabetes, beta cells are able to process and secrete both the mutant and wild-type insulin (2). The cases presented by us and Riar and colleagues indicate that agents reducing the need for endogenous insulin secretion (ie, metformin and SGLT2i) are associated with improvements in blood glucose levels, whereas agents with beta cell stimulating effects are associated with progressive hyperglycemia, suggesting a potential negative impact on beta cell function (3). Although metformin’s mode of action at the level of the beta cell is not fully understood, proposed mechanisms may involve improvement in insulin secretion and protection against pancreatic beta cell apoptosis. Further, metformin improves peripheral insulin sensitivity (8). The SGLT2i increases glucose urine excretion and thereby decreases blood glucose levels and insulin demand as well.

The report has limitations. First, we were not able to account for potential insulin antibodies, which may interfere with the enzyme-linked immunosorbent assay used to measure insulin levels during OGTT. Further, it remains unknown if GlnB22-insulin levels changed before and after noninsulin therapy.

In conclusion, diabetes caused by the heterozygous pathogenic variant R46Q in the insulin gene may be well treated on noninsulin regimens, in particular with agents that reduce the demand for endogenous insulin secretion. Metformin seems to play a key role, and SGLT2 inhibitors can be added when metformin monotherapy is insufficient. This case report indicates long-term durability of the dual treatment regimen on glycemic control. The approach might also be effective for patients with other insulin gene pathogenic variants with sustained beta cell function. However, additional reports or studies are needed to confirm this.

## Learning Points

- Heterozygous missense mutations in the insulin gene are rare causes of MODY, and optimal treatment strategies remain uncertain.
- Diabetes in patients with the heterozygous pathogenic variant R46Q in the insulin gene is probably explained by a combination of lower receptor binding affinity of the mutant insulin and some degree of impaired beta cell function.
- Diabetes caused by this pathogenic variant may be effectively treated with oral antidiabetic agents that lower the need for endogenous insulin secretion (ie, metformin and SGLT2 inhibitor).

## Data Availability

Original data generated and analyzed during this study are included in this published article.
